# Comparison of Gastric versus Gastrointestinal PBET Extractions for Estimating Oral Bioaccessibility of Metals in House Dust

**DOI:** 10.3390/ijerph14010092

**Published:** 2017-01-18

**Authors:** Kristina Boros, Danielle Fortin, Innocent Jayawardene, Marc Chénier, Christine Levesque, Pat E. Rasmussen

**Affiliations:** 1 Environmental Health Science and Research Bureau, HECSB, Health Canada, 50 Colombine Driveway, Tunney’s Pasture 0803C, Ottawa, ON K1A 0K9, Canada; kristina.boros@gmail.com (K.B.); innocent.jayawardene@hc-sc.gc.ca (I.J.); marc.chenier@canada.ca (M.C.); christine.levesque@canada.ca (C.L.); 2 Department of Earth and Environmental Sciences, University of Ottawa, 25 Templeton St., Ottawa, ON K1N 6N5, Canada; Danielle.Fortin@mac.com

**Keywords:** metals, in vitro bioaccessibility, gastric, gastrointestinal, indoor environments, exposure assessment, house dust

## Abstract

Oral bioaccessibility estimates for six metals which are prevalent as contaminants in Canada (zinc, lead, cadmium, copper, nickel, and chromium) are investigated for house dust using the simple gastric phase versus the two-phase physiologically-based extraction technique (PBET). The purpose is to determine whether a complete gastrointestinal (GI) assay yields a more conservative (i.e., higher) estimate of metal bioaccessibility in house dust than the gastric phase alone (G-alone). The study samples include household vacuum dust collected from 33 homes in Montreal, Canada, plus four certified reference materials (NIST 2583, NIST 2584, NIST 2710 and NIST 2710a). Results indicate that percent bioaccessibilities obtained using G-alone are generally greater than or equivalent to those obtained using the complete GI simulation for the six studied metals in house dust. Median bioaccessibilities for G-alone/GI in household vacuum dust samples (*n* = 33) are 76.9%/19.5% for zinc, 50.4%/6.2% for lead, 70.0%/22.4% for cadmium, 33.9%/30.5% for copper and 28.5%/20.7% for nickel. Bioaccessible chromium is above the detection limit in only four out of 33 samples, for which G-alone results are not significantly different from GI results (*p* = 0.39). It is concluded that, for the six studied metals, a simple G-alone extraction provides a conservative and cost-effective approach for estimating oral bioaccessibility of metals in house dust.

## 1. Introduction

There is a growing demand for information on indoor environmental quality, particularly in northern countries such as Canada where residents spend at least 90% of their time indoors [1]. For non-volatile contaminants, such as trace metals, the driving exposure parameter for most health risk assessments at contaminated sites is the assumed rate of ingestion [2]. Thus, soil and household dust have become important sampling media for estimating residential contaminant exposures, most importantly in the case of toddlers and pre-schoolers for whom oral ingestion is a key pathway related to common hand-to-mouth activities [3,4].

The recent focus on human exposures to metals in soil and dust has revealed data gaps that need to be addressed in order to build a better understanding of metal sources and transformations in residential environments [4]. When evaluating human exposures to metals at contaminated sites, risk assessments had traditionally assumed that metals bound to soil are 100% available for absorption by the human gastrointestinal (GI) tract after ingestion [5]. However, considerable research effort has focused on developing and validating suitable standardized extraction methods for estimating human oral contaminant bioaccessibility in vitro, which have shown that where there are insoluble or poorly soluble metal forms (species) in the soil, an assumption of 100% bioavailability may overestimate the health risk [5,6,7,8,9,10,11,12].

Interlaboratory and intermethod comparisons of in vitro approaches for estimating bioaccessibility of metals in soils have established that the pH of the extraction protocol is the key parameter [5,9,12]. The low pH of simulated G-alone extractions (pH 1.2 to 2.5) generally yields higher (more conservative) bioaccessibility estimates than two-phase extractions which conclude with a circum-neutral pH (pH 5.5 to 7.5) to mimic the intestinal environment [5,9,12,13]. The single-phase G-alone extraction has gained regulatory acceptance for lead and arsenic in soil, based on correlations with in vivo bioavailability estimates obtained using swine and/or rodent animal models [10,13,14].

Although bioaccessibility approaches developed for soils are also applicable to house dust [6,15], there are significant differences between house dust and soil matrices which can influence dissolution, most importantly the differences in organic content and metal-organic speciation [16,17,18]. Li et al. [15] showed that house dust, like soil, yields higher Pb bioaccessibility estimates for the G-alone than the intestinal phase. However, some studies suggest that an intestinal phase assay may yield a more conservative estimate of bioaccessibility for other metals in house dust and soil [3,19,20,21,22]. For UK urban soils, Sialelli et al. [23] reported that chromium (Cr), iron (Fe) and, to a lesser extent, copper (Cu) in urban soils showed higher bioaccessibility under intestinal conditions, while lead (Pb) and zinc (Zn) were more bioaccessible in the G-alone phase.

The aim of the present study is to determine how bioaccessibility estimates obtained using the G-alone assay compare to estimates obtained using the complete GI simulation for six metals in house dust including Zn, Pb, cadmium (Cd), Cu, nickel (Ni) and Cr. The physiologically based extraction technique (PBET) developed by Ruby et al. [6] is one of the most widely applied two-phase bioaccessibility assays for risk assessments of contaminated soil, and has been the subject of extensive intermethod comparisons, laboratory round robins, and comparisons with animal models [5,6,7,13,15]. The present study applies the PBET, as adapted for a recent round robin study [5], to a subset of 33 dust samples collected during a residential study in Montreal, Canada [24], and certified reference materials (CRMs) for indoor dust (NIST 2583 and NIST 2584) and soil (NIST 2710 and NIST 2710a). Results from the complete GI simulation are then compared with results from the G-alone assay to determine an appropriate approach for estimating bioaccessibility of the six study metals in house dust.

## 2. Materials and Methods

### 2.1. Samples and Reference Materials

A subset of 33 household vacuum dust samples was selected from a complete set of 225 samples collected during a previous study (September 2009 to March 2010) in Montreal, Canada [24]. Samples were selected on the basis of total concentrations of the six target metals to ensure that variability in metal concentrations across the complete set of Montreal homes was captured in the subset (Table 1). Household vacuum bags were stored frozen in Zip-Lock bags until shipped to Health Canada laboratories for processing. The dust samples were air-dried and sieved to <80 micron according to Canadian House Dust Study protocols described in detail by Rasmussen et al. [25,26]. These six elements were selected because they commonly occur in contaminated sites in Canada [26], and also because published bioaccessibility data are available for these elements from extensive interlaboratory comparison studies [5,9,18]. Certified reference materials (CRMs) were purchased from the National Institute of Standards and Technology (NIST; Gaithersburg, MD, USA): NIST 2583 —Trace Elements in Indoor Dust, NIST 2584—Trace Elements in Indoor Dust (Nominal 1% Lead), NIST 2710a—Montana Soil I, and NIST 2710—Montana Soil.

### 2.2. Physiologically Based Extraction Technique (PBET)

***Gastric (G-alone) Phase.*** To simulate the G-alone phase, an extraction solution was prepared using 1 L deionized water (18.2 MΩ·cm) and adding 1.25 g pepsin, 0.50 g sodium citrate, 0.50 g DL malic acid disodium salt, 420 µL lactic acid (85%), and 0.5 mL/L acetic acid (reagents from Sigma-Aldrich, Oakville, ON, Canada). The pH of the gastric solution was adjusted to 1.8 using the drop-wise addition of high purity HCl, and the solution was heated to 37 °C, after which the pH was checked and adjusted if necessary. A 100 mL aliquot of the gastric solution was added to 125 mL high-density polyethylene (HDPE) plastic bottles containing the pre-weighed dust samples (1.00 ± 0.05 g). The samples were then placed in a pre-heated water bath at 37 °C for 1 h using the end-over-end rotator designed by Drexler and Brattin [10]. A 10 mL aliquot of the extract was then removed, and 1 mL of extract was filtered (using a disposable 0.45 µm cellulose acetate filter syringe with luer-lock tip), into 9 mL of 0.1 HNO_3_ and stored at 4 °C until analysis. House dust samples were extracted in triplicate, and a NIST CRM was included in each batch.

***Gastrointestinal (GI) Phase*.** The remaining gastric digest for each sample or CRM was neutralized to pH 7.0 by adding solid sodium bicarbonate (Sigma-Aldrich, Oakville, ON, Canada) and returned to the pre-heated water bath at 37 °C. The simulated intestinal solution was prepared by dissolving 175 mg bile salts (Sigma-Aldrich, Oakville, ON, Canada) and 50 mg pancreatin (Sigma-Aldrich, Oakville, ON, Canada) in 10 mL deionized water. Once the solutions reached pH 7.0 ± 0.2 at 37 °C (approx. 30 min), a 10 mL aliquot of simulated intestinal solution was added and pH was adjusted if necessary. The digests were then rotated for another four hours to simulate the time it would take to pass through an intestinal system, checking pH after 2 h. A 1 mL aliquot of the GI extract was filtered (0.45 µm) into 9 mL of 0.1 HNO_3_ and stored at 4 °C until analysis.

### 2.3. Instrumental Analysis and Calculation of Percent Bioaccessibility

Metal concentrations were determined in the G-alone and GI digests using an Elan DRC II Axial Field Technology Inductively Coupled Plasma Mass Spectrometer (ICP-MS, Perkin Elmer, Waltham, MA, USA). High purity acids (SEASTAR Chemicals Inc., Sidney, BC, Canada) and ultrapure Milli-Q water (18.2 MΩ·cm) were used for preparation of samples and standards, and high purity standard stock solutions (Delta Scientific Laboratory Products Ltd., Mississauga, ON, Canada) were used to prepare the calibration and internal standard solutions. Equations prescribed by the U.S.-EPA Method 200.8 were used to correct for interferences. Procedural blanks and NIST CRMs were included in each batch. The limit of detection (LOD) was calculated separately for each metal in each phase using three times the standard deviation of 23 procedural blank values. These analyses of the G-alone and GI extracts yielded the “bioaccessible concentrations” of metals in the solid samples (mg/kg). The term “percent bioaccessibility” refers to the fraction (%) of the total metal concentration extracted by the PBET. Percent bioaccessibility was calculated for each sample by dividing the bioaccessible metal concentration (numerator) by the total metal concentration (denominator), and multiplying by 100. Thus, summary statistics (mean, median and percentiles) were based on % bioaccessibilities of individual samples. Total metal concentrations for the Montreal house dust samples were determined by Actlabs Inc. (Ancaster, ON, Canada) using a 4-acid digestion (HF, HClO_4_, HNO_3_, and HCl) followed by Inductively-Coupled Plasma Optical Emission Spectrometry (ICP-OES) and/or Mass Spectrometry. This strong acid digestion was selected in order to obtain quantitative (NIST-traceable) measurements of total metal concentrations in the samples and certified reference materials, as detailed previously [26].

## 3. Results

### 3.1. Comparison of G-Alone and GI Percent Bioaccessibility in NIST CRMs

Results from the PBET extractions (for G-alone and complete GI extractions) are presented in Table 2 for two indoor dust CRMs (NIST 2583 and NIST 2584) and two soil CRMs (NIST 2710 and NIST 2710a). NIST 2710 was included in the present study in accordance with the U.S.-EPA [27] recommendation to include either NIST 2710 or NIST 2711 as a basis for comparison of results from different in vitro bioaccessibility methods. At the time of writing, NIST 2710 is no longer available and has been replaced with NIST 2710a, and therefore both are included in the present study (Table 2).

Differences in reported % bioaccessibilities that arise from using different protocols are mainly attributed to the pH of the extraction fluid, and to a lesser extent, the constituents of the extraction fluids, the means of physical mixing, extraction time, and solid-to-fluid ratios [5,9,12,28]. Another source of variability is the analytical method used to determine the total metal concentration (total concentrations reported on the NIST certificate and/or quantitative strong acid digestions were used in Table 2, whereas some studies may use milder, “quasi-total” extractions for the denominator [29]. In the present study, the more acidic G-alone phase (pH 1.8) resulted in higher Pb bioaccessibility than the circum-neutral GI phase for NIST 2710 (Table 2), consistent with the results of Ellickson et al. [28] who used NIST 2710 to compare saliva + gastric bioaccessibility for Pb (76.1% ± 11%) with a complete saliva + gastric + intestinal extraction for Pb (10.7% ± 2.3%). The difference in G-alone pH between the present study and that used by Ellickson et al. [28] (pH 1.8 compared to pH 1.4, respectively) is likely the main reason for the higher G-alone Pb bioaccessibility for NIST 2710 in the latter study (64.9% compared to 76.1%, respectively). Other studies which use pH 1.5 for the G-alone also report higher Pb bioaccessibilities (mid-70% range) for NIST 2710 [10,18,25] than the present study.

With respect to other metals in NIST 2710, recent studies [5,30] report that the G-alone yields the highest % bioaccessibilities, consistent with the results of the present study (Table 2). Koch et al. [5] reported a significant negative trend with increasing pH for five elements in NIST 2710 (As, Cd, Cu, Pb and Zn), based on results from 17 laboratories using a wide variety of protocols. Using the BARGE UBM in vitro method, Wragg and Cave [30] also reported that the G-alone yielded the highest % bioaccessibility for Cd, Pb and As in NIST 2710. Sequential extraction techniques were used to identify which solid phase fractions of NIST 2710 were the most bioaccessible under gastric conditions, and revealed that the carbonate and exchangeable phases were the likely hosts of the bioaccessible Cd in NIST 2710, while bioaccessible Pb was divided amongst carbonate, exchangeable, and oxide phases [30].

Table 2 indicates that the G-alone phase returned higher % bioaccessibilities than the GI phase for both house dust CRMs (NIST 2583 and NIST 2584). Differences between G-alone and GI values were significant (*p* < 0.01) for all metals except for Cu in NIST 2583 (*p* = 0.06) and Cu in NIST 2584 (*p* = 0.07). This study is the first to report GI results for the two indoor dust CRMs (Table 2), and thus, comparisons with the literature are possible only for the G-alone phase. G-alone % bioaccessibilities for Pb in NIST 2583 and NIST 2584 using pH 1.8 in the present study (31.1% and 56.1% respectively, Table 2) were lower by >30% compared to studies using pH 1.5, which range from 65% to 83% for Pb in NIST 2583 and from 81% to 91% for Pb in NIST 2584 [18,25,29]. Synchrotron X-ray analysis indicated the presence of two common Pb paint pigments in NIST 2584: predominately Pb carbonates (with gastric bioaccessibilities of 73% to 76%) and lesser amounts of Pb chromate (with only 9% gastric bioaccessibility) [31,32]. Bioaccessible Cr was below LOD in all NIST CRMs (Table 2). G-alone values for Cu in the present study (Table 2) were 20% to 30% lower than G-alone values reported by Dodd et al. [18] using pH 1.5 (53% for Cu in NIST 2583 and 73% for Cu in NIST 2584). Likewise, G-alone values for Ni in the present study (Table 2) were 10% to 15% lower than G-alone values reported by Dodd et al. [18] using pH 1.5 (42% for Ni in NIST 2583 and 34% for Ni for NIST 2584). As is the case with Pb, these comparisons indicate that the G-alone % bioaccessibilities for Cu and Ni in the indoor dust CRMs are strongly influenced by the pH used in the protocol. 

G-alone values for Zn and Cd in the two indoor dust CRMs do not appear to be as strongly influenced by differences in pH among protocols. G-alone values for Zn in the present study (Table 2) were similar to those reported by Dodd et al. [18] using pH 1.5 (92% for Zn in NIST 2583 and 93% for Zn in NIST 2584). G-alone values for Cd (Table 2) were within the range reported by Le Bot et al. [29] and Dodd et al. [18] (69% to 94% for NIST 2583 and 76% to 95% for NIST 2584), despite differences in pH conditions.

### 3.2. Variability of Total and Bioaccessible Metals in House Dust Samples

Total concentrations for Cd, Cu, Ni and Cr in the Montreal vacuum samples (Table 1) are comparable to national baseline estimates from the Canadian House Dust Study (median values of 3.5 mg/kg for Cd; 199 mg/kg for Cu; 62.3 mg/kg for Ni, and 99 mg/kg for Cr; *n* = 1025 [26]. Total Pb is elevated in the Montreal samples (median 176 mg/kg; Table 1) compared to the national baseline (median 100 mg/kg for Pb; *n* = 1025) [26]. Elevated Pb concentrations in these homes may be related to their location in four of the oldest boroughs of Montreal, where Pb-based paint and Pb service lines are potential residential Pb sources [24]. Total Zn is also elevated in the Montreal samples (median 879 mg/kg; Table 1) compared to the national baseline (median 725 for Zn; *n* = 1025), which is another characteristic of older homes [26].

Zn and Cd are both readily solubilized in acidic environments, and displayed the highest G-alone % bioaccessibilities in the Montreal samples (median values of 76.9% and 70.0% respectively; Table 3), to the extent that Zn and Cd were completely extracted from the dust in the G-alone phase in a few samples, within experimental error (i.e., 100% ± 10% bioaccessibility). These results are consistent with the international range of house dust bioaccessibility values (80% to 90% for Zn, and 50% to 90% for Cd) compiled by Ibanez et al. [33]. In the case of Cd, soil studies have suggested that the G-alone phase may over-predict bioavailability due to the high solubility of Cd in the acidic solution [9,34]. For G-alone Zn values, it was necessary to use the Limit of Quantification (LOQ; 10 times standard deviation (SD) of the procedural blank; Table 3) as the quality criterion (instead of LOD) to handle outliers (>110% bioaccessibility) as described previously [31]. 

Median G-alone Pb bioaccessibility in the Montreal dust samples (50.4%; *n* = 33; Table 3) is lower than that reported for the Canadian House Dust Study (CHDS; 59%; *n* = 1025) [25], likely due to the lower gastric pH used in the CHDS (pH 1.5). Median G-alone bioaccessibilities for Cu (33.9%) and Ni (28.5%) in the Montreal samples were also considerably lower than values reported for Ottawa for Cu (46%) and Ni (41%) based on a pH 1.5 extraction [17]. Ibanez et al. [33] found that reported bioaccessibilities for Cu and Ni are highly variable in house dust, ranging from 20% to 80%. In the Montreal samples, Cr displayed the lowest bioaccessibility of all the metals, with only four samples displaying bioaccessible Cr above LOD (Table 3). These values are within the international range of 10% to 50% compiled by Ibanez et al. [33] for Cr bioaccessibility in house dust. The large number of house dust samples containing Cr below LOD (29 out of 33; Table 3) is consistent with soil studies which report low Cr bioaccessibility due to the low solubility of common Cr species [35,36]. 

Using the data in Table 1 to calculate coefficients of variation (COV = mean/sd × 100) for total metal concentrations, a high degree of variability in dust metal contamination among the Montreal homes can be observed. COVs range from 130% to 186% for total Cd, Cr and Pb, and from 77% to 86% for total Cu, Zn and Ni (Table 1). However, the variability of percent bioaccessibility from home to home, based on COVs derived from Table 3, tends to be lower. COVs for Cd and Cu bioaccessibility are within 30% to 40% for both G-alone and GI, while COVs for Ni bioaccessibility are within 40% to 50% for both G-alone and GI. COVs for Zn and Pb bioaccessibility are much lower in the G-alone phase (22% and 34% respectively) compared to the GI phase (105% and 115% respectively), which may indicate an analytical source of variability in the GI phase. The COV for Cr bioaccessibility is quite high for both G-alone and GI (106% and 91% respectively). Variability in metal bioaccessibility amongst different homes has been attributed primarily to variability in metal speciation: Rasmussen et al. [25] reported a significant positive relationship (*R*^2^ = 0.85) between gastric Pb bioaccessibility in house dust samples predicted using XANES speciation and Pb bioaccessibility measured in the same samples using a gastric extraction. Synchrotron-based studies have attributed the heterogeneity of metal compounds in house dust to the fact that there are both indoor and outdoor sources [25,37,38,39], and have shown that Pb speciation is particularly variable in older homes [40].

### 3.3. Comparison of G-Alone and GI Percent Bioaccessibility in House Dust Samples

**Figure 1:**
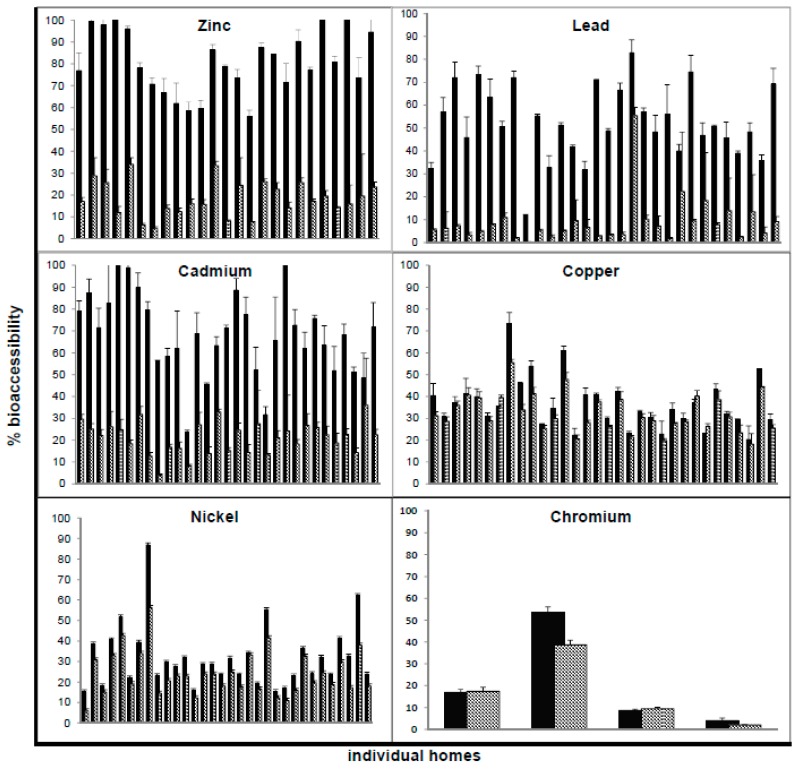
Percent bioaccessibility results for house dust samples comparing gastric (dark bars) and gastrointestinal (light bars) extractions. Bars indicate average of triplicate digests for each home; error bars indicate standard deviation. A total of 33 homes were studied; only results above LOD are shown.

G-alone versus GI values for the six studied metals are displayed for individual homes using bar graphs in Figure 1. Overall, the G-alone extraction yielded higher % bioaccessibilities than the complete GI extraction for Zn, Pb, Cd, Cu, and Ni (*p* < 0.01), assessed using paired *t*-tests of all samples where bioaccessibility exceeded detection/quantification limits using both phases (for Zn *n* = 25; Pb *n* = 30; Cd *n* = 31; Ni *n* = 32; and Cu *n* = 32). The only metal that did not display a significant difference between the G-alone and GI phases for house dust samples above LOD was Cr (*p* = 0.39; *n* = 4). In the Cr bar graph (Figure 1), the two samples on the right represent the highest total Cr concentrations in the study (393 and 868 ppm respectively), with relatively low G-alone/GI bioaccessibilities of 8.7%/9.4% and 4.7%/2.1% respectively. The two samples on the left in the Cr bar graph (Figure 1) contained lower Cr concentrations (154 and 74 ppm respectively) but higher bioaccessibilities, with G-alone/GI results of 17.0%/17.5% and 52.8%/38.7% respectively.

With respect to the other five metals in Figure 1 (Zn, Pb, Cd, Cu, Ni), comparisons of G-alone vs. GI for individual homes showed that G-alone results were higher than or equal to GI results in all homes, with the exception of Cu in two out of 33 homes. In these two homes, G-alone/GI bioaccessibilities were 35.0% ± 0.5%/39.6% ± 1.1% and 22.6% ± 0.6%/26.3% ± 1.2%; although the GI results were only a few percentage points above G-alone; in both cases the difference was significant (*p* < 0.01).

**Figure 2:**
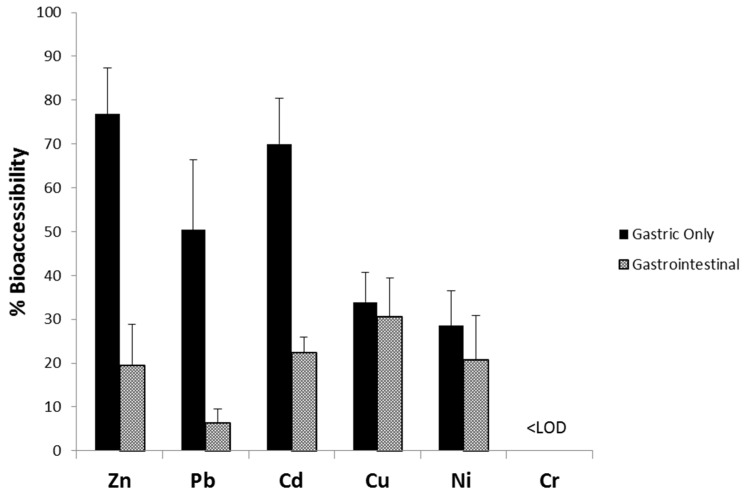
Comparison of median bioaccessibility values from the house dust samples (*n* = 33) using the PBET G-alone versus GI method. Error bars show the 75th percentiles.

Figure 2, which summarizes the median % bioaccessibilities for G-alone and GI extractions of the house dust samples, illustrates the decrease in % bioaccessibility from stomach to intestine for all metals (Cr not included; median <LOD for both phases). These results are consistent with previous studies that have shown that metal bioaccessibility is higher in the stomach phase than in the intestinal phase mainly due to the higher pH in the intestinal phase which causes re-adsorption and precipitation of metals [5,6,7,9]. For example, Li et al. [15] compared four different two-phase bioaccessibility methods for Pb in house dust, including the PBET. All four methods yielded lower values from the intestinal phase compared to the G-alone, which the authors attributed to the higher pH of the intestinal phase causing (1) co-precipitation of Pb with Fe and (2) re-adsorption of Pb onto the dust matrix [15].

## 4. Discussion

Previous reports that PBET yields higher GI% bioaccessibility results than other GI assays made it an appropriate end-member assay for the purpose of this study, which was to determine whether the complete GI assay yielded a more conservative (higher) estimate of metal bioaccessibility in house dust than G-alone. Of the four bioaccessibility methods compared by Li et al. [15], PBET returned the highest Pb % bioaccessibility in the intestinal phase, which was attributed to sodium citrate remaining in solution from the G-alone and inhibiting the co-precipitation of Fe and Pb in the intestinal phase. As PBET was the only assay that used sodium citrate, the authors concluded this component was the cause for higher intestinal phase results for PBET compared to other methods [15].

The experimental results (Figure 1 and Table 3) showed that G-alone values were higher than or equivalent to GI values for all six metals using PBET, with the exception of Cu in two out of 33 homes. Greater Cu bioaccessibility in the intestinal phase than the G-alone has been observed previously for both soil and dust [3,19,20,22,23,35]. As the pH rises, Cu appears to be stabilized in solution by complexation with available organic ligands from the dust matrix, and with other reagents used in the extraction procedure such as the malate ion and anions of bile acids [19]. An intermethod comparison study by Li et al. [41] found that the digestive enzymes used in the PBET gastric phase kept soil Cu in solution during the intestinal phase, despite the increase in pH. Their study showed that the solubility of soil Cu in the intestinal phase of PBET remained constant or was even promoted as a result of the presence of the digestive enzymes, whereas the solubility of soil Zn and Pb was pH dependent [41].

Although the observation of higher Cu bioaccessibility in the GI extraction compared to the G-alone extraction (for two out of 33 house dust samples) is consistent with the above published studies of Cu complexing behaviour in GI simulations, the difference was only a few percentage points. Compared to the addition of an intestinal phase extraction, the selection of pH conditions for the gastric phase had a much greater and more ubiquitous impact on Cu bioaccessibility. As indicated earlier, the use of pH 1.8 in the G-alone PBET phase yielded lower estimates of bioaccessibility by >30 percentage points for Cu and Pb and by 10–15 percentage points for other metals, compared to published values for the same CRMs using G-alone protocols at pH 1.5. Therefore, concerns about lower G-alone Cu bioaccessibility results could be addressed by selecting a G-alone protocol with lower pH conditions.

The results show that, overall, the complete GI assay does not yield a more conservative estimate of metal bioaccessibility in house dust compared to G-alone for the studied metals. As explained by Koch et al. [5], the observation that G-alone yields higher or similar results to the complete GI assay does not negate the potential usefulness of the intestinal phase. In fact, Juhasz et al. [34] reported that for Cd in soil, the PBET intestinal phase showed a better correlation with in vivo results than the gastric phase, despite lower bioaccessibilities. In comparing the median bioaccessibilities for different metals in Figure 2, it is notable that the order of decreasing bioaccessibility is different for the GI extraction compared to the G-alone extraction. For the G-alone phase, the order of decreasing bioaccessibility was Zn > Cd > Pb > Cu > Ni > Cr (Figure 2). In contrast, for the GI phase, the order of decreasing bioaccessibility was Cu > Cd > Ni > Zn > Pb > Cr. This difference reflects the greater tendency of certain metals (especially Cu) to form stable organic complexes in the gastric phase that remain in solution as the pH rises during the intestinal phase, in contrast with metals that are primarily controlled by pH. Since the G-alone versus GI extractions provide different information, for certain applications the selection of an appropriate assay may be metal-dependent. Also, the observation that two homes in the present study displayed GI > G-alone for Cu, in combination with the wide variability in metal bioaccessibilities described in the literature and shown in Table 3 (especially for Cr), highlights the importance of site-specific approaches to improve human health risk assessments for exposure to contaminated soils and dust.

## 5. Conclusions

The results show with statistical significance that, overall, the G-alone extraction yields more conservative (higher) or equally conservative estimates of bioaccessibility for the metals Zn, Pb, Cd, Cu, Ni and Cr in house dust, compared to the two-stage GI extraction. It is concluded that, for the studied metals, a single-stage simulation of the gastric phase provides a conservative and cost-effective approach for estimating oral bioaccessibility of ingested metals in house dust.

## Figures and Tables

**Table 1:** Statistical summary of total metal concentrations (mg/kg) in the complete set of house dust samples from 225 Montreal homes and the subset of 33 house dust samples selected for Physiologically Based Extraction Technique (PBET) extractions in the present study (<80 µm fraction). SD = Standard deviation; LOD = Limit of detection.

Metal	Complete Set (*n* = 225)			Subset Used for PBET (*n* = 33)		
	Mean (SD)	Range	Median	Mean (SD)	Range	Median
Zn	979 (610)	27.6–5190	879	1177 (1007)	27.6–5190	982
Pb	415 (838)	6.2–8000	176	962 (1787)	6.2–8000	195
Cd	4.4 (4.4)	<LOD–38.6	3.2	6.0 (7.8)	<LOD–38.6	2.9
Cu	278 (413)	4.7–5810	205	266 (204)	4.7–1020	213
Ni	80.7 (145)	2.4–2120	61.2	78.6 (67.0)	2.4–313	60.0
Cr	82.6 (73.6)	1.4–868	67.1	117 (152)	1.4–868	86.4

**Table 2:** Percent bioaccessibility of metals (mean ± standard deviation) in certified reference materials for the gastric (G-alone) and gastrointestinal (GI) PBET extraction phases. Total concentrations are certified values reported in the Certificates of Analysis unless indicated otherwise.

Sample	Extraction	Zn	Pb	Cd	Cu	Ni	Cr
NIST 2710 (*n* = 3)	Total (mg/kg)	6952 ± 91	5532 ± 80	21.8 ± 0.2	2950 ± 130	14.3 ± 1	39 ^a^
	G-alone (%)	28.8 ± 1.3	64.9 ± 1.7	74.4 ± 2.1	63.3 ± 0.8	14.0 ± 0.4	<LOD
	GI (%)	11.4 ± 1.0	15.7 ± 0.6	39.3 ± 2.5	44.6 ± 2.0	9.6 ± 1.2	<LOD
NIST 2710a (*n* = 6)	Total (mg/kg)	4180 ± 150	5520 ± 30	12.3 ± 0.3	3420 ± 50	8 ± 1	23 ± 6
	G-alone (%)	42.3 ± 1.8	45.7 ± 1.4	45.6 ± 1.5	53.0 ± 1.8	12.8 ± 0.7	<LOD
	GI (%)	12.1 ± 0.6	4.9 ± 0.2	22.7 ± 0.7	37.2 ± 0.6	8.2 ± 0.5	<LOD
NIST 2583 (*n* = 5)	Total (mg/kg)	896 ± 56.7 ^b^	85.9 ± 7.2	7.3 ± 3.7	233 ± 19.4 ^b^	93.9 ± 8.4 ^b^	80 ± 22
	G-alone (%)	95.3 ± 2.6	31.1 ± 1.1	69.7 ± 5.2	33.0 ± 1.0	26.4 ± 2.4	<LOD
	GI (%)	42.7 ± 1.8	13.4 ± 0.8	39.6 ± 3.2	31.8 ± 0.8	21.4 ± 1.1	<LOD
NIST 2584 (*n* = 5)	Total (mg/kg)	2580 ± 150	9761 ± 67	10 ± 1.1	288 ± 29.4 ^b^	84.6 ± 10.1 ^b^	135 ± 9.1
	G-alone (%)	84.7 ± 1.6	56.1 ± 1.7	80.1 ± 3.8	43.4 ± 0.27	23.9 ± 0.5	<LOD
	GI (%)	21.1 ± 0.6	7.6 ± 0.2	28.9 ± 1.2	42.3 ± 1.24	18.7 ± 0.8	<LOD

^a^ Provisional values provided by NIST; ^b^ from Rasmussen et al. [26].

**Table 3:** Bioaccessible metal concentration (mg/kg) and percent bioaccessibility for house dust samples from 33 Montreal homes (<80 µm fraction), comparing results for the gastric (G-alone) and gastrointestinal (GI) PBET extraction phases (SD = Standard deviation; LOD = Limit of detection).

Metal	Gastric Phase					Gastrointestinal Phase				
	LOD	Mean (SD)	Median	95th Percentile	# Samples >LOD *	LOD	Mean (SD)	Median	95th Percentile	# Samples >LOD *
**Zn**										
Conc. (mg/kg)	191 ^++^	955 (872)	750	2325	27	71.1	214 (216)	162	485	28
% Bioaccessibility		76.1 (16.4)	76.9	100			28.3 (29.7)	19.5	109	
**Pb**										
Conc. (mg/kg)	3.2	578 (1192)	96.1	3006	31	0.4	55.6 (111)	15.2	238	32
% Bioaccessibility		51.6 (17.6)	50.4	77.8			8.4 (9.7)	6.2	20.0	
**Cd**										
Conc. (mg/kg)	0.1	4.3 (5.6)	2.1	17.1	33	0.1	1.3 (1.6)	0.7	4.8	32
% Bioaccessibility		70.7 (22.0)	70.0	105			20.7 (7.8)	22.4	32.3	
**Cu**										
Conc. (mg/kg)	2.6	91.2 (72.2)	71.3	253	32	1.3	80.5 (57.0)	72.9	211	33
% Bioaccessibility		36.1 (11.5)	33.9	56.7			34.0 (12.9)	30.5	50.9	
**Ni**										
Conc. (mg/kg)	0.8	21.6 (19.1)	16.5	52.9	32	0.9	15.6 (12.2)	12.4	42.4	32
% Bioaccessibility		31.3 (15.2)	28.5	58.0			23.9 (10.6)	20.7	42.0	
**Cr**										
Conc. (mg/kg)	20	<LOD	<LOD	40.6	4	18	<LOD	<LOD	36.3	4
% Bioaccessibility		20.9 (22.2)	12.8	47.9			17.4 (15.9)	14.5	35.8	

* For results below LOD, a value of 0.5 LOD was substituted except for Cr (calculation of % bioaccessibility for Cr based on four homes >LOD). ^++^ LOQ for Zn (637 mg/kg) also considered as G-alone quality criterion.
